# Antibacterial Activity of Leaf Extracts of *Baeckea frutescens* against Methicillin-Resistant *Staphylococcus aureus*

**DOI:** 10.1155/2014/521287

**Published:** 2014-06-16

**Authors:** Somayeh Razmavar, Mahmood Ameen Abdulla, Salmah Binti Ismail, Pouya Hassandarvish

**Affiliations:** Department of Molecular Medicine, Faculty of Medicine, University of Malaya, Malaysia

## Abstract

This study was based on screening antibacterial activity of the ethanol extract of *Baeckea frutescens* L. against MRSA clinical isolates, analyzes the potential antibacterial compound, and assesses the cytotoxicity effect of the extract in tissue culture. Leaves of *Baeckea frutescens* L. were shade dried, powdered, and extracted using solvent ethanol. Preliminary phytochemical screening of the crude extracts revealed the presence of alkaloids, flavonoids, steroids, terpenoids, phenols, and carbohydrates. The presence of these bioactive constituents is related to the antibacterial activity of the plant. Disc diffusion method revealed a high degree of activity against microorganisms. The results confirm that *Baeckea frutescens* L. can be used as a source of drugs to fight infections caused by susceptible bacteria.

## 1. Introduction

In recent years, there has been an increasing awareness about the importance of medicinal plants. Drugs from these plants are easily available, inexpensive, safe, efficient, and rarely accompanied by side effects. Plants which have been selected for medical use over thousands of years constitute the most obvious starting point for new therapeutically effective drugs such as anticancer drugs [1] and antimicrobial drugs [2]. Recently, medicinal plants usage has increased in spite of the advances made in the field of chemotherapy. The reasons proposed [3] are the use of medicinal plants as materials for the extraction of active pharmacological agents or as precursors for chemicopharmaceutical hemisynthesis. There is also the increased use of medicinal plants in industrialized countries for galenic preparations and herbal medicines.

*Baeckea frutescens* L. of the family Myrtaceae and subfamily Myrtoideae is a medicinal plant that has an essential oil which has been used as a traditional drug in South East Asia.* Baeckea frutescens* L. is a small tree found in mountainous areas of South China, Hong Kong, South East Asia, and Australia. The local Malay name of this plant is “Cucur Atap.” The needle-like leaves are small and narrow in only about 6–15 mm long. When crushed, the leaves give off a resinous aromatic fragrance. The tiny fruits split, releasing minute angular seeds. Tea made from these leaves is used to treat fever in China [4]. It is one of the traditional folk medicine in Indonesia [4]. Packets of leaves are burned under the bed of colic sufferers.

The essential oil has been used for aromatherapy and is inhaled for mental clarity and to ease mental distress [5]. The oil is also used when massaging aching muscles and to treat pain on the surface of the body in addition to its use as a bath or tonic [5].

This paper presents a preliminary phytochemical investigation of* Baeckea frutescens* L., which is responsible for the antibacterial activity of the extracts of the leaves on methicillin-resistant* Staphylococcus aureus* (MRSA) bacterial species.

## 2. Materials and Methods

### 2.1. Preparation of Plant Extracts

Test plant was first collected from the Rimba Ilmu, University of Malaya. All parts of the plant except the roots were oven-dried at 56°C for several days until fully dried and then ground to fine powder with a blender machine. The extraction was done at room temperature. The powder was soaked in absolute ethanol at a 1 : 20 ratio for 7 days and then filtered by Whatman filter paper number 1. The filtrate was collected and evaporated under vacuum using the BUCHI Switzerland Rotary Evaporator to obtain concentrated, powdered extracts. All extracts were stored at 4°C for further use. The ranged yield of extracts is 5–20% (w/w).

### 2.2. Bacterial Culture

A bacterial culture is a method of bacteria organisms by allowing them to reproduce in predetermined culture media under controlled laboratory conditions. For any bacterial culture, it is necessary to provide the suitable environmental and nutritional conditions that exist in its natural habitat.

The methicillin-resistant* Staphylococcus aureus* (MRSA) pure isolates used in this study were kindly provided by Professor Dr. Yassim of the Microbiology Laboratory of University Malaya Medical Centre. The streak plate method is the most common way of separating bacterial cells on the agar surface.

Confirmation of the identity of working strains was done by colony morphology and gram staining as described in the Textbook of Diagnostic Microbiology [6]. The bacterial isolate was maintained in Brain Heart Infusion (BHI) agar (Pronadisa, Spain) slants at 4°C.

### 2.3. Disc Diffusion Method

Disc diffusion method was used for antibacterial activity. A stock solution of extract was prepared by dissolving 0.1 g of extract with 100 mL of their respective solvents (distilled water and absolute ethanol) to produce a final concentration of 100 mg/mL. The stock solution was then diluted to concentrations of 2.5, 5, 10, 20, 50, and 100 mg/mL of extract. 20 *μ*L of each dilution was impregnated into sterile, blank discs 6 mm in diameter. 5 *μ*L of extract was spotted alternately on both sides of the discs and allowed to dry before the next 5 *μ*L was spotted to ensure precise impregnation. Distilled water and ethanol-loaded discs were used as negative controls for aqueous and ethanol extracts, respectively. All discs were fully dried before the application on bacterial lawn. The positive controls used were vancomycin antibiotic discs (Becton-Dickinson, USA) for all* S. aureus* strains. Antibacterial activity was evaluated by measuring the diameter of the inhibition zone (IZ) around the discs. The assay was repeated trice. Antibacterial activity was expressed as the mean zone of inhibition diameters (mm) produced by the leaf extract.

### 2.4. Column Chromatography (CC) Spectral Analysis

The sample is dissolved in a solvent and applied to the front of the column (wet packing) or alternatively adsorbed on a coarse silica gel (dry packing). Using a ratio of 100 g of silica gel/g of crude sample allows for relatively easy separation. The solvent elutes the sample through the column, allowing the components to separate. The ethanol soluble phase was subjected to silica gel column chromatography using AcOH-MeOH (90 : 10) solvent system.

### 2.5. HPLC Analysis

A HPLC test was performed using an Agilent Zorbax column (Xdb-C18 Type MG 5 *μ*m, 4.6 × 250 mm). The detection wavelengths were 200, 230, 254, and 320 nm. Elution was carried out with CH_3_CN-H_2_O at the flow rate of 1.2 mL/min. The injection volume was 100 *μ*m. Samples were mixed and vacuum dried to 29.6 mg. Then the samples were dissolved in 1.0 mL of distilled water. A stock solution (12,00 ppm) was prepared by adding 405.4 *μ*L of sample solution (29.6 mg/mL) to 594.6 *μ*L of distilled water, which was kept refrigerated at 4°C. The samples were filtered using a SRP-4 membrane 0.45 *μ*M before they were injected into HPLC. The fractions were collected and subjected to profiling.

### 2.6. Liquid Chromatography-Mass Spectrometry

Liquid chromatography-mass spectrometry (LC-MS, or alternatively HPLC-MS) is an analytical chemistry technique that combines the physical separation capabilities of liquid chromatography (or HPLC) with the mass analysis capabilities of mass spectrometry. LC-MS is a powerful technique used for many applications which has very high sensitivity and selectivity.

2 mg of sample was prepared by dissolving in 2 mL methanol in volumetric flask. Solution was then filtered by using SRP-4 membrane 0.45 mm. Stock solution 1 mg/mL was kept in fridge at 4°C.

LCMS was performed with an Acquity BEH C18, 2.1 × 50 mm, 1.7 *μ*m UPLC columns. Elution was carried out with %H20 + 0.1% F.A at the flow rate 0.5 mL/min. The injection volume was 3 *μ*L.

## 3. Results and Discussion

Phytochemical screening of the crude extracts of* Baeckea frutescens* L. revealed the presence of flavonoids and phenolic compounds (Table 1).

The presence of alkaloid is interesting as significant quantities are used as antimalarial, analgesics, and stimulants [7]. Flavonoids, which are known to prevent tumor growth and also used to protect against gastrointestinal infections, are of pharmacognostic importance thus lending credence to the use of the plant in ethnomedicine [8]. Some of these bioactive compounds that are synthesized as secondary metabolites as the plant grows are also used to protect the plant against microbial attacks and predation by animals [8].

According to the results of disc diffusion assay, this plant has active compounds that are effective for the prevention of infections caused by MRSA.

There are a number of factors that could influence the results of the disc diffusion assay. Firstly, the diameter of the zones is affected by the rate of diffusion of the antimicrobial compound [9, 10] and thus may not exactly represent the potency of the extract's antimicrobial activity. Where studies of plant extracts are concerned, the disc preparation technique could present with another problem wherein the extract was not properly and evenly impregnated into the paper discs. Another important factor is the standardization of the inoculum size to 0.5 McFarland turbidity. This inoculum size is important to ensure confluent or almost confluent lawn growth as a smaller inoculum size (such that single colonies are seen) may produce falsely large inhibition zones while a bigger inoculum size (thick bacterial lawn) may produce falsely smaller zones instead [11].

The ethyl acetate, methanol, and acid acetic solvents were more effective than other solvents to show inhibition zone against MRSA (Table 2).

The zone produced by the plant extract against the MRSA was from acid acetic and methanol solvents were the largest zone. The lowest zone of growth inhibition was ethyl acetate and hexane.

**Figure 1:**
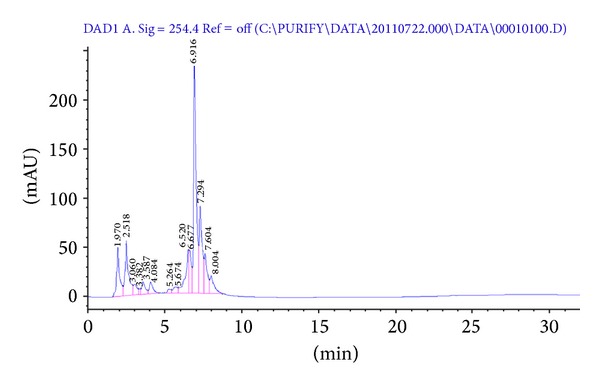
HPLC profiling of samples by UV 254 nm. Indicating the compounds shown in Figure 2.

To determine the chemical constituents of the biological activity in ethyl acetate : normal-hexane and acid acetic : methanol soluble phases, HPLC analysis was performed (Figure 1).

One principal peak and several lesser peaks were observed in the ethyl acetate (EtOAc): normal-hexane soluble phases. Compound 1 was isolated from the EtOAc soluble phase by repeated column chromatography on silica gel.

**Figure 2:**
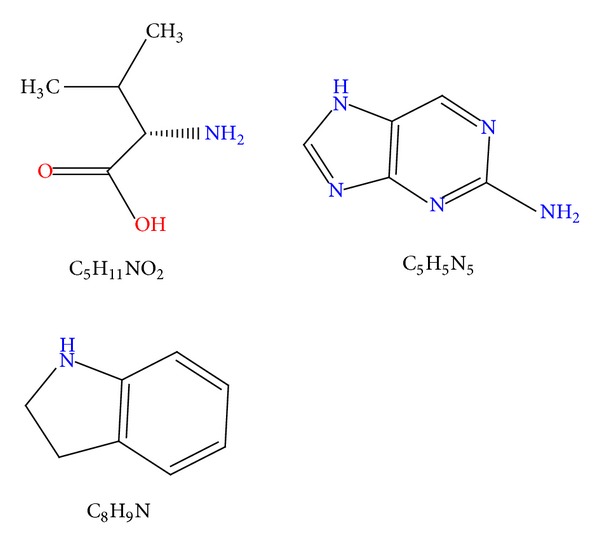
Chemical structures of compounds.

The molecular formula of the main peak was determined to be C_5_H_11_NO_2_, C_5_H_5_N_5_ or C_8_H_9_N by liquid chromatography-mass spectrometry (Table 3 and Figure 2).

Results obtained for antibacterial activity against MRSA for 3 main peaks were in Table 4.

From the results of the disc diffusion screening,* B. frutescens* is shown to clearly possess antibacterial properties against MRSA. As* B. frutescens* seems to give appreciable antibacterial activity against all gram-positive staphylococcal strains, this may indicate that the plant extract acts specifically against the gram-positive cell wall, particularly the staphylococcal cell wall [12] because they have a much thicker peptidoglycan layer than gram-negative bacteria. This outer membrane is composed of lipopolysaccharides that give gram-negative bacteria extra resistance against antibiotics that cannot penetrate it, for example, glycopeptides like vancomycin [13].

Antibacterial activity of ethanol extract of* B. frutescens* leaf has been assessed by measuring the diameters of zones of growth inhibition on some strain of bacteria and the results are presented as shown in Table 5.

Inhibition growth of the highest zone has been shown by ethanol extract against gram-positive bacteria like MRSA (14.5 mm),* Staphylococcus aureus* (13 mm), and* Bacillus* (9.5 mm). The growth inhibition was moderately active against gram-negative bacteria* Escherichia coli* (8.5 mm) and* Klebsiella* (0 mm).

## 4. Conclusion

The result of this study showed that* Baeckea frutescens* L. extract contains phytochemical components. Potentially, these compounds have the most important applications against human pathogens, including those that cause enteric infections. The results of various screening tests indicate that the leaves have some measurable inhibitory action against gram-positive bacteria such as* Staphylococcus aureus* (MRSA).

## Figures and Tables

**Table 1:** Result of the phytochemical screening of ethanol extracts of leaves of *Baeckea frutescens* L.

S. number	Phytochemical compounds	Leaves of crude extracts
1	Flavonoids	+
2	Glycosides	+
3	Phenolic	+

**Table 2:** Antibacterial activity of ethanol extract of *Baeckea frutescens *in different solvents against MRSA.

S. number	Zone of inhibition (mm) against MRSA				Solvents in CC
	Rep. 1	Rep. 2	Rep. 3	Mean	
1	10	8	12	10	Ethyl acetate and hexane
2	20	22	18	20	
3	16	16	16	16	
4	20	18	22	20	
5	6	8	4	6	
6	14	10	18	14	
7	8	6	10	8	
8	16	18	14	16	
9	10	10	10	10	
10	15	10	20	15	

1	0	0	0	0	Acid acetic and methanol
2	10	12	18	10	
3	30	25	35	30	

Ethyl ACETATE	0	0	0	0	Control
Hexane	0	0	0	0	
Acid acetic	0	0	0	0	
Methanol	0	0	0	0	

**Table 3:** Liquid chromatography-mass spectrometry.

Mass [M + H]	Molecular formula [M]	Number of hits [M]
118.0874	C_5_H_11_N_O2_	665
136.0623	C_5_H_5_N_5_	140
120.082	C_8_H_9_N	148

**Table 4:** Antibacterial activity of liquid chromatography-mass spectrometry results against MRSA.

Number	Molecular formula [M]	Zone of inhibition (mm) against MRSA			
		Rep. 1	Rep. 2	Rep. 3	Mean
1	C_5_H_11_N_O2_	10	11	10	10.33
2	C_5_H_5_N_5_	11	12	12	11.66
3	C_8_H_9_N	10	10	10	10

**Table 5:** Zone of inhibition against some bacteria strains by ethanol extract of *Baeckea frutescens *L.

Zone of inhibition (mm)						
Bacteria strain	Conc. of extract (mg/mL)				Gram	Shape
	100	50	20	10		
MRSA ST/0903-24	14.0	11.5	9.5	7.5	Positive	Cocci
MRSA ST/0904-25	12.0	8.5	7.0	—	Positive	Cocci
MRSA ST/0904-28	14.5	11.5	8.0	7.5	Positive	Cocci
MRSA ST/0904-30	12.0	8.5	—	—	Positive	Cocci
*S.aureus *	13.0	11.5	7.5	—	Positive	Cocci
*E.coli *	8.5	7.5	7.0	7.5	Negative	Bacilli
*Klebsiella* sp.	—	—	—	—	Negative	Diplococci
*Bacillus* sp.	9.5	8.5	7.0	7.0	Positive	Bacilli
*P*. *aeruginosa *	—	—	—	—	Negative	Bacilli
